# Sexual Function of Patients with Deep Endometriosis after Surgical Treatment: A Systematic Review

**DOI:** 10.1055/s-0043-1772596

**Published:** 2023-11-29

**Authors:** Graziele Vidoto Cervantes, Paulo Augusto Ayroza Galvão Ribeiro, Mariana Carpenedo Tomasi, Daniela Farah, Helizabet Salomão Abdalla Ayroza Ribeiro

**Affiliations:** 1 Department of Gynecology, Endometriosis and Laparoscopic Surgery Center, Faculdade de Ciências Médicas da Santa Casa de São Paulo, São Paulo, SP, Brazil; 2 Department of Gynecology, Women's Health Technology Assessment Center, Escola Paulista de Medicina, Universidade Federal de São Paulo, São Paulo, SP, Brazil

**Keywords:** systematic review, endometriosis, sexual health, surgery, dyspareunia, revisão sistemática, endometriose, saúde sexual, cirurgia, dispareunia

## Abstract

**Objective** To review the current state of knowledge on the impact of the surgical treatment on the sexual function and dyspareunia of deep endometriosis patients.

**Data Source** A systematic review was conducted in accordance with the Meta-Analysis of Observational Studies in Epidemiology (MOOSE) guidelines. We conducted systematic searches in the PubMed, EMBASE, LILACS, and Web of Science databases from inception until December 2022. The eligibility criteria were studies including: preoperative and postoperative comparative analyses; patients with a diagnosis of deep endometriosis; and questionnaires to measure sexual quality of life.

**Study Selection** Two reviewers screened and reviewed 1,100 full-text articles to analyze sexual function after the surgical treatment for deep endometriosis. The risk of bias was assessed using the Newcastle-Ottawa scale for observational studies and the Cochrane Collaboration's tool for randomized controlled trials. The present study was registered at the International Prospective Register of Systematic Reviews (PROSPERO; registration CRD42021289742).

**Data Collection** General variables about the studies, the surgical technique, complementary treatments, and questionnaires were inserted in an Microsoft Excel 2010 (Microsoft Corp., Redmond, WA, United States) spreadsheet.

**Synthesis of Data** We included 20 studies in which the videolaparoscopy technique was used for the excision of deep infiltrating endometriosis. A meta-analysis could not be performed due to the substantial heterogeneity among the studies. Classes III and IV of the revised American Fertility Society classification were predominant and multiple surgical techniques for the treatment of endometriosis were performed. Standardized and validated questionnaires were applied to evaluate sexual function.

**Conclusion** Laparoscopic surgery is a complex procedure that involves multiple organs, and it has been proved to be effective in improving sexual function and dyspareunia in women with deep infiltrating endometriosis.

## Resumo

**Objetivo** Revisar a literatura publicada sobre o impacto do tratamento cirúrgico na função sexual e na dispareunia de pacientes com endometriose profunda.

**Fonte de Dados** Uma revisão sistemática foi realizada de acordo com as diretrizes Meta-Analysis of Observational Studies in Epidemiology (MOOSE). Realizamos pesquisas sistemáticas nas bases de dados PubMed, EMBASE, LILACS e Web of Science desde o início até dezembro de 2022. Os critérios de elegibilidade foram estudos que incluíam: análises comparativas pré- e pós-operatórias; pacientes com diagnóstico de endometriose profunda; e a aplicação de questionários para avaliar a função sexual.

**Seleção dos Estudos** Dois revisores selecionaram e revisaram 1.100 artigos para analisar a da função sexual após o tratamento cirúrgico da endometriose profunda. O risco de viés foi calculado usando-se a escala de Newcastle-Ottawa para estudos observacionais e a ferramenta para ensaios clínicos randomizados da Cochrane Collaboration. O estudo foi cadastrado no International Prospective Register of Systematic Reviews (PROSPERO; cadastro CRD42021289742).

**Coleta de dados** Variáveis gerais sobre os estudos, a técnica cirúrgica, os tratamentos complementares e os questionários foram inseridas em uma planilha do Microsoft Excel 2010 (Microsoft Corp., Redmond, WA, Estados Unidos).

**Síntese dos dados** Foram incluídos 20 estudos em que se usou a técnica de videolaparoscopia para a excisão da endometriose profunda. Uma meta-análise não pôde ser realizada devido à heterogeneidade substancial entre os estudos incluídos. As classes III e IV da escala revisada da American Fertility Society foram predominantes, e múltiplas técnicas cirúrgicas foram usadas para o tratamento da endometriose. Questionários padronizados e validados foram aplicados para avaliar a função sexual.

**Conclusão** A cirurgia laparoscópica é um procedimento complexo que envolve múltiplos órgãos, e provou ser eficaz na melhora da função sexual e da dispareunia em mulheres com endometriose profunda.

## Introduction

Endometriosis is defined as the presence of endometrial stroma and glands outside the uterine cavity. It is present in 3% to 15% of fertile women, 1 and it affects women's quality of life, causing chronic pelvic pain, dyspareunia, infertility, as well as certain deleterious sexual effects in 67% of the cases. 2 In contrast, deep infiltrating endometriosis (DIE) consists of the penetration of the endometrial tissue more than 5 mm below the peritoneal surface. 3

The literature reports that endometriotic disease is the main cause of dyspareunia, and it affects 60% to 70% of women undergoing surgery. The common presence of DIE on cardinal and uterosacral ligaments, on the pouch of Douglas and on the posterior vaginal fornix represents a nine-old increase in the risk of developing dyspareunia. 2 4

Dyspareunia does not cause only pain: it is also associated with psychological and psychosocial injury. Feelings of fear during intercourse, as well as guilt, are predominant among DIE patients, and they directly and indirectly affect domains of sexual function such as desire, frequency, pleasure and orgasm. 5

The treatment for endometriosis is mainly focused on pain control and quality of life improvement, including, sexual life. Hormonal therapies are effective for pain control during disease progression, but they can also lead to gonadal suppression and reduced sexual response. 6 However, surgical procedures and radical resection of all visible endometriosis nodules may improve quality of life in up to 85% to 95% of severe to moderate cases. 7

According to international guidelines, endometriosis is a chronic disease that requires a life-long management plan to control pain symptoms and to avoid multiple surgical procedures. 8 Hormonal therapies to achieve a hypoestrogenic status are effective to control pain and disease progression, but they are also associated with gonadal suppression and reduced sexual response. 6 The aim of the surgical treatment is the excision of all endometriosis lesions to improve pain and infertility. However, in cases of extensive DIE, surgery is associated with peri- and postoperative complications, as well as a decrease in sexual function. 9

Thus, the present systematic review aims to assess how surgery affects sexual function and dyspareunia in patients undergoing surgical treatment to treat DIE.

## Materials and Methods

The present systematic review was conducted in accordance with the Meta-Analysis of Observational Studies in Epidemiology (MOOSE) guidelines. The study protocol was registered at the at the International Prospective Register of Systematic Reviews (PROSPERO; registration CRD 42021289742) and followed the Preferred Reporting Items for Systematic Reviews and Meta-Analyses (PRISMA) statement. 10

We performed a search in the following databases: PubMed, EMBASE, Cochrane Library, LILACS, and Web of Science from inception to December 2022. The main keywords used were *deep endometriosis* , *sexual function* , *resection* , and *shaving* . The full search strategy used can be found in Chart 1 .

**Chart 1:** Searchstrategy for the selection of studies

Database	Search Strategy	Number Of Studies
PubMed	( *deep endometriosis* OR *deep infiltrating endometriosis* OR *endometrioma* ) AND ( *resection* OR *excision* OR *nodulectomy* OR *cystectomy* OR *shaving* OR *rectosigmoidectomy* ) AND *(dyspareunia OR (sexual AND (function OR quality OR behavior) OR (pain OR dysfunction) AND (sexual OR sexual intercourse)*	313
EMBASE	( *deep endometriosis/exp* OR *deep endometriosis* OR *deep infiltrating endometriosis/exp* OR *deep infiltrating endometriosis* OR *endometrioma/exp* OR *endometrioma* ) AND ( *resection/exp* OR *resection* OR *excision/exp* OR *excision* OR *nodulectomy/exp* OR *nodulectomy* OR *cystectomy/exp* OR *cystectomy* OR *shaving/exp* OR *shaving* OR *rectosigmoidectomy/exp* OR *rectosigmoidectomy* ) AND *dyspareunia OR (sexual AND (function OR quality OR sexual behavior) OR (pain OR dysfunction) AND (sexual OR sexual intercourse) AND (article/it OR article in press/it OR review/it) AND [female]*	597
Cochrane Library	( *deep endometriosis* OR *deep infiltrating endometriosis* OR *endometrioma* ) AND ( *resection* OR *excision* OR *nodulectomy* OR *cystectomy* OR *shaving* OR *rectosigmoidectomy* ) *AND (dyspareunia OR (sexual AND (function OR quality OR sexual behavior) OR (pain OR dysfunction) AND (sexual OR sexual intercourse)*	20
LILACS	( *deep endometriosis* OR *deep infiltrating endometriosis* OR *endometrioma* ) AND ( *resection* OR *excision* OR *nodulectomy* OR *cystectomy* OR *shaving* OR *rectosigmoidectomy* ) AND ( *dyspareunia* OR *(sexual AND (function OR quality OR sexual behavior) OR (pain OR dysfunction) AND (sexual OR sexual intercourse)*	9
Web of Science	( *deep endometriosis* OR *deep infiltrating endometriosis* OR *endometrioma* ) AND ( *resection* OR *excision* OR *nodulectomy* OR *cystectomy* OR *shaving* OR *rectosigmoidectomy* ) AND ( *dyspareunia* OR *(sexual AND (function OR quality OR sexual behavior)) OR (pain OR dysfunction) AND (sexual OR sexual intercourse)*	161

Two independent reviewers (GC and DF) were invited to analyze all articles found. Initially, an analysis of the titles and abstracts was performed to screen for potential eligible studies. Later, the reviewers evaluated the fully screened articles to select eligible studies. Disagreements were resolved by joint review and consensus among reviewers.

To comply with the objectives of the present systematic review, the eligibility criteria were as follows: comparative studies on female sexual function before and after surgery for deep endometriosis; studies with women previously diagnosed with deep endometriosis by physical examination or complementary imaging exams submitted to surgery; and studies with the application of standardized questionnaires to assess sexual function and dyspareunia. No clinical treatment associated with surgery was established, neither a limited time of follow-up after surgery, nor were there language restrictions during the initial search. The exclusion criteria were: conference abstracts, case reports, case series, reviews, and duplicate studies. In the full-text analysis, articles published in languages other than English, Portuguese, Italian, Spanish, and French were also excluded.

The two reviewers (GC and DF) inserted the data from all the included studies in a Microsoft Excel 2010 (Microsoft Corp., Redmond, WA, United States) spreadsheet. We extracted general variables form the studies, such as authorship, year of publication, country, type of study, follow-up, surgery performed, age of the patients, and the number of patients included. We also recorded the name of the questionnaire used for the evaluation of sexual function and dyspareunia. The heterogeneity among the studies and questionnaires found in the literature did not enable the performance of a meta-analysis.

The outcome of interest was the assessment of sexual function before and after surgery using a validated questionnaire. The presence of dyspareunia before and after the surgery was also evaluated.

To evaluate the risk of bias in non-randomized studies (such as case-control and cohort studies), we used the Newcastle-Ottawa Scale (NOS), while the risk of bias in randomized controlled trials (RCT) was evaluated using the Cochrane Collaboration's tool (RoB-1). 11 12

The NOS is based on a star scoring system in which the observational study is assessed in terms of three broad parameters: selection of the study groups; comparability of the groups; and ascertainment of either the exposure or the outcome of interest for case-control or cohort studies respectively. 11 On the other hand, the RoB-1 covers six domains of the possible biases of RCTs: selection bias, performance bias, detection bias, attrition bias, reporting bias, and other biases. Each domain is classified as low, high, or unclear risk of bias. 12

## Results

**Fig. 1:**
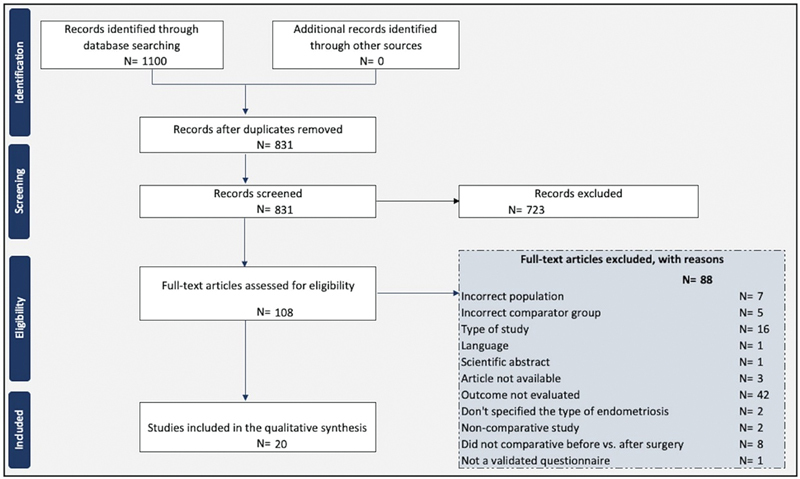
Flowchart o the search and selection of studies.

We found 1,100 studies; after removing the duplicates, 831 studies were screened for titles and abstracts by 2 reviewers who selected 108 studies for full-text analyses. Finally, a total of 20 studies fulfilled the eligibility criteria and were included in the present systematic review. A flowchart of the search and selection of studies is summarized in Fig. 1 .

Observational studies and one RCT were included in the review. Half of the cohort studies (50%) had a score ≥ 7 stars on the NOS scale, while 38% had 6 stars, and 2, ≤ 5 stars. The RCT had a score of 6 stars on the NOS scale; it was on a comparison of laparoscopic surgeries with and without uterosacral ligament resection, and it presented an unclear risk of bias for random sequence generation and allocation sequence concealment, and a high risk for blinding of the outcome assessment. In total, the studies included evaluated 2,145 patients with follow-ups ranging from 3 to 69 months. The characteristics of the included studies are presented in Chart 2 .

**Chart 2:** Characteristics of the studies selected

Author, year	Country	Type of study	N	Type of surgery	Age in years	Sexual function questionnaire	Dyspareunia questionnaire
** Garry et al., 27 2000 **	United Kingdom	Prospective	57	Laparoscopic excision surgery	–	SAQ	NRS
** Abbot et al., 24 2003 **	Australia	Prospective	254	Laparoscopic excision surgery	Median: 31 (range 20–48)	SAQ	VAS
** Vercellini et al., 32 2003 **	Italy	Randomized controlled trial	180	Laparoscopic excision surgery	Mean 30 ± 5	SSRS	VAS
** Ferrero et al., 26 2007 **	Italy	Prospective	98	Laparoscopic excision surgery	Mean 34.6 ± 3.4	DSFI; GSSI	–
** Ferrero et al., 25 2007 **	Italy	Prospective	73	Laparoscopic excision surgery	Mean 34.7 ± 4.3	DSFI; GSSI	VAS
** Meuleman et al., 15 2009 **	Belgium	Retrospective	56	Laparoscopic excision surgery with CO _2_ laser	Median:32 (range: 24–42)	SAQ	VAS
** Meuleman et al., 13 2012 **	Belgium	Retrospective	45	Laparoscopic excision surgery with CO _2_ laser	Median 30 (range: 18–42)	SAQ	VAS
** Mabrouk et al., 33 2012 **	Italy	Prospective	125	Laparoscopic excision surgery	Mean 35.4 ± 5.5	SHOW-Q	VAS
** Setälä et al., 16 2012 **	Finland	Prospective	22	Laparoscopic excision surgery or combined laparoscopic vaginal surgery	Median: 29 (range: 19–40)	MFSQ	VAS
** Kossi et al., 21 2013 **	Finland	Prospective	26	Laparoscopic excision surgery	Median: 33.5 (range: 22–46)	MFSQ	–
** Van den Broeck et al., 14 2013 **	Belgium	Prospective	203 (total); 76 WB; 127 WOB	Laparoscopic excision surgery with CO _2_ laser	–	SSFS	–
** Di Donato et al., 31 2015 **	Italy	Prospective	250 DIE; 250 HG	Laparoscopic excision surgery	DIE: mean 34 ± 5 HG: mean 32 ± 6	SHOW-Q	–
** Fritzer et al., 17 2016 **	Germany	Prospective	96	Laparoscopic excision surgery or combined laparoscopic vaginal surgery	Median: 30.8 (range: 18–45)	FSDS; FSFI	NRS
** Pontis et al., 18 2016 **	Italy	Prospective	16	Combined transurethral and laparoscopicd surgeries	Mean: 29.12 ± 4.33	FSFI	–
** Riiskjaer et al., 20 2016 **	Denmark	Prospective	128	Laparoscopic excision surgery	Mean: 33.8 ± 5.3	SVQ	1: never; 2: a little; 3: often; 4: very often
** Uccella et al., 29 2018 **	Italy	Prospective	34	Laparoscopic excision surgery	Median 39 (range: 27–51)	FSFI	–
** Lermann et al., 19 2019 **	Germany	Retrospective	134 WOB; 113 WB; 100 CG	Laparoscopic excision surgery	WOB: mean 34.3 ± 6; WB: mean – 37.7 ± 6.	KFSP	–
** Ianieri et al., 28 2022 **	Italy	Retrospective	100	Laparoscopic Excision Surgery	Mediana:38 (32,5–43)	FSFI	VAS
** Martínez-Zamora et al., 34 2021 **	Spain	Prospective	193 (total); 129 DIE; 64 CG	Laparoscopic excision surgery	DIE: mean 33.5 ± 6.04; CG: mean 34.7 ± 4.5	SQoL-F; FSDS; B-PFSF	–
** Zhang et al., 30 2022 **	China	Retrospective	55	Laparoscopic excision surgery	Mean: 30 ± 3	FSFI	–

Abbreviations: B-PFSF, Brief Profile of Female Sexual Function; CG, control group; CO _2_ , carbon dioxide; DIE, deep infiltrating endometriosis; DSFI, Derogatis Sexual Functioning Inventory; FSDS, Female Sexual Distress Scale, revised; FSFI, Female Sexual Function Index; GSSI, Global Sexual Satisfaction Index; HG, healthy group; KFSP, Kurzfragebogen Sexualität und Partner-schaft; MFSQ, McCoy Female Sexuality Questionnaire modified by Wiklund et al; NRS, Numeric Rating Scale; SAQ, Sexual Activity Questionnaire; SSFS, Short Sexual Functioning Scale; SHOW-Q, Sexual Health Outcomes in Women Questionnaire; SQoL-F, Sexual Quality of Life − Female Questionnaire; SQV, Sexual Function-Vaginal Changes Questionnaire; SSRS, Sabbatsberg Sexual Rating Scale; VAS, Visual Analogue Scale; WB, with bowel resection; WOB, without bowel resection.

A comparison of the pre- and postoperative outcomes regarding sexual function and dyspareunia is shown in Chart 3 .

**Chart 3:** Preoperative and postoperative comparison of sexual function and dyspareunia according to the questionnaires applied

		Sexual Function			Dyspareunia		
Autor, year	Follow-up (months)	Preoperatively	Postoperatively	Significance	Preoperatively	Postoperatively	Significance
**Questionnaire: SAQ**							
Garry et al., 27 2000	4	Pleasure: 11 (6 ± 13)	Pleasure: 13 (9 ± 16)	Pleasure: 0.002	7 (5.5 ± 9)	0 (0 ± 4)	0.0001
		Discomfort: 3 (1.5 ± 5)	Discomfort: 1 (0 ± 3)	Discomfort: < 0.05			
		Habit:1 (0 ± 1)	Habit:1 (1 ± 2)	Habit: < 0.002			
Abbott, et al., 24 2003	60	Pleasure:10 (5 ± 12)	Pleasure:12 (9 ± 16)	Pleasure: 0.001	Median: 6.0 (0.0–9.0)	0.0 (0.0–4.0)	< 0.001
		Discomfort: 3 (1 ± 5)	Discomfort: 2 (1.5 ± 3)	Discomfort: < 0.012			
		Habit:1 (0 ± 1)	Habit:1(1 ± 1)	Habit:0.001			
Meuleman et al., 15 2009	29	–	–	Pleasure: < 0.0001	5 (0–10)	1 (0–10)	< 0.0001
				Discomfort: < 0.0001			
				Habit< 0.0001			
Meuleman et al., 13 2012	27	–	–	Pleasure: 0.009	28 (0–95)	1 (0–63)	< 0.0001
				Discomfort: 0.026			
				Habit: 0.0003			
**Questionnaire: FSFI**							
Pontis et al., 18 2016	12	26 ± 2.5	28 ± 1.7	< 0.001	–	–	–
Uccella et al., 29 2018	6	19.1 (1.2–28.9)	22.7 (12.2–31)	0.004	–	–	–
Ianieri et al., 28 2022	3	P: 19.4 ± 9.8	P: 21.6 ± 10.8	0.34	P: 5.2 ± 3.6	P: 0.9 ± 2.2	< 0.001
		NP 23.8 ± 3.7	NP: 23.7 ± 8.1		NP: 3.7 ± 3.5	NP: 0.1 ± 0.5	
Zhang et al., 30 2022	26	26.1 ± 3	26.8 ± 3	0.25	–	–	–
**Questionnaire: FSFI and FSDS**							
Fritzer et al., 17 2016	10 FSFI	–	–	DIE: 0.21	DIE: 6.18	DIE: 2.49	< 0.001
				Vaginal: 0.98			
				Peritoneal: 0.11	Vaginal: 6.64	Vaginal: 2.18	< 0.001
	FSDS	–	–	DIE: 0.04			
				Vaginal: 0.25	Peritoneal: 5.05	Peritoneal: 2.85	< 0.001
				Peritoneal: 0.34			
**Questionnaire: SHOW-Q**							
Mabrouk et al., 33 2012	6	Satisfaction: 51	Satisfaction: 65	< 0.0005	7 ± 3	1 ± 3	< 0.0001
		Orgasm: 57	Orgasm: 59	0.7			
		Desire: 55	Desire: 64	< 0.0004			
Di Donato et al., 31 2015	12	Satisfaction: 50	Satisfaction: 75	< 0.001	–	–	–
		Orgasm:63	Orgasm:62	Not significant			
		Desire: 58	Desire: 72	< 0.001			
**Questionnaire: DSFI and GSSI**							
Ferrero et al., 26 2007	3 DSFI	Frequency with USL: 1.3 ± 0.7; without USLE: 1.6 ± 0.7	Frequency with USL: 2.3 ± 0.7; without USL: 2.2 ± 0.8	Frequency ith USL: < 0.001; without USL: 0.004	–	–	–
	3 DSFI	Orgasm with USL: 2.3 ± 1.0; without USL: 2.9 ± 1.0	Orgasm with USL: 4.4 ± 1.1; without USL: 3.1 ± 1.5	Orgasm with USL: 0.001; without USL: 0.003			
	3 GSSI	With USL: 3.4 ± 1.7; without USL: 4.1 +/− 1.7	With USL: 5.5 ± 1.9; without USL: 5.3 +/− 1.8	With USL: 0.001; without USL: 0.003			
Ferrero et al., 25 2007	6 DSFI	Frequency with USL: 1.1 ± 0.6; without USL: 1.3 ± 0.9	Frequency with USL: 1.8 ± 0.8; without USL: 2.2 ± 1.1	Frequency with USL: < 0.001; without USL:< 0.001	With USL: 7.6 ± 1.1; without USL: 7.1 ± 1.0	With USL: 2.8 ± 1.9; without USL: 2.4 ± 1.8	< 0.001
	6 DSFI	Orgasm with USL: 2.3 ± 1.2; without USL: 3.1 ± 1.0	Orgasm with USL: 1.3 ± 0.9; without USL: 4.2 ± 1.3	Orgasm with USL: < 0.001; without ULSE: < 0.003			
	6 GSSI	With USL: 3.2; without USL: 3	With USL: 5; without USL: 5.8	< 0.001 < 0.001			
	12 DSFI	Frequency with USL: 1.1 ± 0.6; without USL: 1.3 ± 0.9	Frequency with USL: 1.9 ± 0.7; without USL: 2.2 ± 1.1	Frequency with USL: < 0.001; without USL: < 0.027	With USL: 7.6 ± 1.1; without USL: 7.1 ± 1.0	With USL: 2.8 ± 2.2; without USL: 2.2 ± 1.8	< 0.001
	12 DSFI	Orgasm with USL: 2.3 ± 1.2; without USL: 3.1 ± 1.0	Orgasm with USL: 1.9 ± 0.7; without USL: 4.0 ± 1.0	Orgasm with USL: < 0.001; without USL: < 0.118			
	12 GSSI	With USL: 3.2; without USL: 3	With USL: 5.2; without USL: 5.6	< 0.001 < 0.001			
**Questionnaire: MFSQ**							
Setälä et al., 16 2012	12	Sexual satisfaction: 21.1	Sexual satisfaction: 2.1	< 0.05	4.3	1.7	< 0.05
		Sexual problem: 6.3	Sexual problem: 1.4	< 0.05			
		Partner satisfaction: 12.1	Partner satisfaction: 0.8	Not significant			
Kossi et al., 21 2013	12	Sexual satisfaction: 20.1	Sexual satisfaction: 2.8	< 0.01	–	–	–
		Sexual problem: 7	Sexual problem: 1.1	< 0.10			
		Partner satisfaction: 12.1	Partner satisfaction: 0.7	< 0.10			
**Questionnaire: KFSP**							
Lermann et al., 19 2019	69	WB: 24	WB: 25	0.416	–	–	–
		WOB: 27.5	WOB: 19.5	0.001			
**Questionnaires: SQOL, FSDS and B-PFSF**							
Martínez-Zamora et al., 34 2021	36	SQOL-F: 70	SQOL-F: 77	< 0.001	–	–	–
		FSDS: 17	FSDS: 10	< 0.001			
		B-PFSF: 18	B-PFSF: 25	< 0.001			
**Questionnaire: SQV**							
Riiskjaer et al., 20 2016	12	Satisfaction: 3 (1–7)	Satisfaction: 4 (1–7)	0.0001	3 (1–4)	2 (1–4)	< 0.0001
		Frequency: 2 (1–5)	Frequency: 3(1–5)	0.0004			
		Desire: 2 (1–4)	Desire: 2 (1–4)	0.0003			
**Questionnaire: SFSS**							
Van den Broeck et al., 14 2013	6	Orgasm – WB:10.5%; WOB:16.3%	Orgasm – WB: 0%; WOB: 10%	< 0.01	WB: 44.8%; WOB: 31.3%	WB: 10.4%; WOB: 12.7%	> 0.05
		Excitation – WB:21.6%; WOB:11.5%	Excitation – WB:7.4%; WOB:13%%	> 0.05			
		Desire – WB:31.7%; WOB: 28.4%	Desire – WB:9.4%; WOB:19.4%	> 0.05			
	18	Orgasm – WB:16.3%; WOB:10,5%	Orgasm – WB: 6.3%; WOB: 2.9%	> 0.05	WB: 44.8%; WOB: 31.3%	WB: 6.3%; WOB: 20%	> 0.05
		Excitation – WB: 21.6%; WOB: 11.5%	Excitation – WB: 6.3%; WOB: 2.9%	> 0.05			
		Desire – WB: 28.4%; WOB: 31.7%	Desire – WB: 12.1%; WOB: 5.7%	> 0.05			
**Questionnaire: SSRS**							
Vercellini et al., 32 2003	18	USL:45.4 ± 19.9	USL:53.8 ± 18.8	0.763	USL: 58 (45–72)	USL: 22 (0–35)	0.0001
		CG: 44.7 ± 20.8	CG: 55.4 ± 15.6		CG: 54 (26–67)	CG: 18 (0–30)	0.0001

Abbreviations: B-PFSF, Brief Profile of Female Sexual Function; CG, control group; DIE, deep infiltrating endometriosis; DSFI, Derogatis Sexual Functioning Inventory; FSDS, Female Sexual Distress Scale, revised; FSFI, Female Sexual Function Index; GSSI, Global Sexual Satisfaction Index; KFSP, Kurzfragebogen Sexualität und Partner-schaft; MFSQ, McCoy Female Sexuality Questionnaire modified by Wiklund et al; NP, no parametrial group; P, parametrial group; SAQ, Sexual Activity Questionnaire; SFSS, Short Sexual Functioning Scale; SHOW-Q, Sexual Health Outcomes in Women Questionnaire; SQoL-F, Sexual Quality of Life − Female Questionnaire; SQV, Sexual Function-Vaginal Changes Questionnaire; SSRS, Sabbatsberg Sexual Rating Scale; USL, uterosacral ligament; WB, with bowel resection; WOB, without bowel resection.

The predominant surgical technique used to treat DIE patients was laparoscopic surgery. A total of 14 articles used only the laparoscopy technique for DIE excision, while 3 studies associated it with the CO _2_ laser technique. 13 14 15 Two studies performed vaginal surgery associated with the laparoscopic procedure, when necessary, 16 17 and one combined laparoscopy with transurethral surgery. 18

In one study, 18 transurethral and laparoscopic surgeries to resect bladder endometriosis presented a significancy improvement in sexual function in all 6 domains of the Female Sexual Function Index (FSFI), with a postoperative score of 28.2 +/− 1.7. Setälä et al. 16 and Fritzer et al. 17 performed vaginal surgery associated with videolaparoscopy procedures to resect vaginal endometriosis lesions, resulting in a significant increase on sexual comfort and pleasure according to the modified McCoy Female Sexuality Questionnaire (MFSQ). 16 However, the study by Fritzer et al. 17 did not show significant results in the final FSFI score in any of the three population groups compared (DIE, vaginal resection, and peritoneal endometriosis). 17 Sexual function after the CO _2_ laser technique was evaluated by two different questionnaires. 13 14 15 The Sexual Activity Questionnaire (SAQ) showed significant postoperative improvement on the following pillars of sexual function: pleasure, habit 13 15 and discomfort. 15 The Short Sexual Function Scale (SSFS) only presented significant improvement in the pillar of orgasm after surgery. 14

Other articles also evaluated sexual function and DIE of the bowel. A comparative study 19 analyzed sexual function for the following sixty-nine months after DIE surgery with and without bowel resection. Postoperatively, the patients without bowel resection improved significantly in all categories on the Kurzfragebogen Sexualität und Partner-schaft (KFSP) questionnaire. Not only no significant postoperative improvement was observed in the patients in the bowel endometriosis group, but this group had significantly poorer scores in comparison with the control group. 19 Riiskjaer et al. 20 performed laparoscopy for DIE of the bowel and observed positive results on the Sexual Function-Vaginal Changes Questionnaire (SQV) after one year of follow-up: there was a significant increase in vaginal changes, general sexual satisfaction, desire for sexual intercourse, and frequency of sexual intercourse. Laparoscopic resection for bowel endometriosis also resulted in an increase in sexual satisfaction on the overall MFSQ score one year after surgery in one study. 21 Sexual problems and satisfaction with partner scores did not change significantly in another study. 22

The surgical data related to the female sexual function response in the studies analyzed were collected and presented in Chart 4 .

**Chart 4:** Surgical data as reported by the studies selected

Author, year	Histological analysis	Endometriosis classification	Intraoperative classification	Nerve-sparing technique	Procedures	Other endometriosis location (%)	Retro cervical (%)	USL (%)	Rectovaginal septum (%)	Vagina (%)	Bowel (%)
** Garry et al., 27 2000 **	No	rAFS	III: 63.2%	No	Complication: 1,9% – bruises	Ovaries: 40.3%; total pouch of Douglas obliteration: 30.4%; partial pouch of Douglas obliteration: 33.3%	33.3%	No Specific side: 77.2%	59.6%	38.52%	56.1%
** Abbot et al., 24 2003 **	Yes	rAFS	I:14%; II: 28%; III: 17%; IV: 41%	No	Complication: 0.3% – iatrogenic bowel injury; 0.6% – transfusion; 0.3% –vaginal deiscense	Total pouch of Douglas obliteration: 32%; partial pouch of Douglas obliteration: 18%; bilateral endometrioma: 12%; right: 18%; left: 12%	–	Unilateral 88%; bilateral: 57%	–	6%	–
** Vercellini et al., 32 2003 **	No	rAFS	I: 39%; II: 22%; III: 20%; IV: 19%	No	–	–	–	No specific side: 100%	–	–	–
** Ferrero et al., 26 2007 **	Yes	–	–	No	–	–	–	No specific side: 65.3%	–	–	–
** Ferrero et al., 26 2007 **	Yes	rAFS	IV-III: 86.9%; II-I: 12.32%	No	–	–	–	No specific side: 64.7%	–	–	–
** Meuleman et al., 15 2009 **	Yes	rAFS	II: 2.22%; III: 4.44%; IV: 95%	Yes	Oophorectomy: 9%; appendectomy: 14%; salpingectomy: 30%; cystectomy: 39%; ureterolysis: 86%; adhesiolysis: 100%; complication: 3.5% – vascular anastomosis; 5.3% – compartmental syndrome	–	11%	–	–	–	Anterior bowel resection: 36%; sigmoid resection: 39%
** Meuleman et al., 13 2012 **	Yes	rAFS	III: 2%; IV: 98%	Yes	Oophorectomy 2%; bladder suture: 7%; appendectomy: 9%; salpingectomy: 38%; cystectomy: 42%; ureterolysis: 91%; complication: 2.2% – transitory urinary retention	–	16%	–	–	–	Sigmoid resection: 90%
** Mabrouk et al., 33 2012 **	Yes	–	–	Yes	Complications: 0.8% – vascular injury; 1.6% –transfusion; 4% – transitory urinary retention; 1.6% – retovaginal fistula; 0.8% – ureterovaginal fistula	55%	–	72%	–	25%	Sigmoid resection: 17%; shaving: 30%
** Setälä et al., 16 2012 **	No	rAFS	–	No	Appendicectomy: 14%; urinary bladder resection: 14%; salpingectomy: 14%; adhesiolysis: 100%; complications: 14% – transitory urinary retention; 4.5% – anemia; 4% – vaginal deiscense	Pouch of Douglas obstruction 7%; peritoneal lesions: 68%	95%	14%	86%	100%	50%
** Kossi et al., 21 2013 **	Yes	–	–	No	Resection of urinary bladder: 7%; appendectomymy: 11%; salpingectomy: 26%; ureterolysis 80%; adhesiolysis: 100%; complications:11.5% – transitory urinary retention; 3.8% – bowel bleeding	Peritoneal lesions: 53%	–	No specific side: 88%	–	61%	100%
** Van den Broeck et al., 14 2013 **	Yes	rAFS	III: 33%; IV: 66%	Yes	–	–	–	–	–	–	100%
** Di Donato et al., 31 2015 **	Yes	–	–	No	–	–	–	–	–	–	–
** Fritzer et al., 17 2016 **	Yes	rAFS	I: 28%; II: 21%; III: 26%; IV: 25%	No	–	Peritoneal lesions: 41%; DIE: 59%	–	–	–	37%	–
** Pontis et al., 18 2016 **	Yes	–	–	No	–	Bladder: 100%	–	–	–	–	–
** Riiskjaer et al., 20 2016 **	No	–	–	No	–	–	–	–	–	–	100%
** Uccella et al., 29 2018 **	No	Enzian	A1 B2 C3 (20.6%); A2 B2 C3 (26.5%); A3 B1 C1 (2.9%); A3 B2 C1 (5.9%); A3 B3 C1 (2.9%); A3 B3 C2 (5.9%); A3 B1 C0 FB (5.9%); A0 B3 C2 FA (5.9%); A3 B1 C1 FA (17.6%); A3 B1 C2 FA (2.9%); A3 B1 C1 FO (2.9%)	Yes	Bilateral adnexectomy/castration: 8.8%; ureterolysis: 100%; complications: 17.6% – transitory urinary retention	–	–	–	–	50%	47.1%
** Lermann et al., 19 2019 **	No	Enzian	–	No	–	WOB: 75.3%; WB: 72.4%	–	Unilateral – WOB: 48.3%; WB:8%; bilateral – WOB: 27%; WB: 24.1%	WOB: 89.9%; WB:87.4%	WOB: 41.6%; WB: 75.9%	WB: 74.33%
** Ianieri et al., 28 2022 **	Yes	rAFS	II: 2.9%; III: 43.5%; IV: 53.6%	Yes	Complications: 1% – hemoperitoneum; 2% – iatrogenic bowel injury	–	48%	–	–	15%	64%
** Martínez-Zamora et al., 34 2021 **	Yes	–	–	No	–	Endometriomas – bilateral: 11.62%; left: 24.8%; right: 13.95%; ureter (no specific side): 24%; bladder: 28.68%; peritoneal lesions: 76%	47.28%	No specific side: 68.99%	11.62%	8.52%	39.53%
** Zhang et al., 30 2022 **	Yes	rAFS	I + II: 20%; III + IV: 35%	No	–	–	–	No specific side: 25.45%	43.63%	–	18%

Abbreviations: DIE, deep infiltrating endometriosis; rAFS, revised American Fertility Society classification; USL, uterosacral ligament; WO, with bowel resection; WOB, without bowel resection.

The extension of the endometriosis was ascertained intraoperatively using the revised American Fertility Society (rAFS) 22 and the Enzian scale 23 in 13 studies. 13 14 15 16 17 19 24 25 26 27 28 29 30 In the evaluated articles, 45.32% of the patients were classified as rAFS class IV (severe), followed by 27.67% as class III (moderate),13.65% as class II (mild), and 13.40% as class I (minimal). The most common pelvic sites of DIE involvement were: the uterosacral ligaments (51.24%), the bowel (31.56%), the vagina (14.45%), the rectovaginal septum (8.89%) and the retrocervical nodule (6.46%). 14 19 20 21 25 26 28 29 30 31

Three comparative studies 25 26 32 evaluated sexual function after resection of the uterosacral ligament. In two of them, 25 26 the authors used the Derogatis Sexual Functioning Inventory (DSFI) and Global Sexual Satisfaction Index (GSSI) to analyze sexual function 6 and 12 months postoperatively, and found a significant increase in sexual function up to 6 months. Frequency and orgasm on the DSFI were not significant at the 12-month follow-up. 25 26 Similar results were presented by Vercellini et al. 32 after 18 months of follow-up, with no significant improvement in sexual function on the Sabbatsberg Sexual Rating Scale (SSRS).

An improvement in sexual function was also observed on FSFI scores after resection of bladder endometriosis, 18 as well as a significant improvement in sexual satisfaction and intercourse pain on the MFSQ after twelve months of surgery in a group of women with DIE submitted to vaginal nodule resection. 16

The nerve-sparing surgical technique for DIE excision was described as necessary in six articles, 13 14 15 28 29 33 in which different results were found: two studies 15 29 showed a significant improvement on the SAQ and the FSFI's global sexual function score; two other studies 13 33 reported partial improvement in some domains on the FSFI and on the Sexual Health Outcomes in Women Questionnaire (SHOW-Q); and the two remaining studies 14 28 reported no difference in sexual response after the nerve-sparing surgery. Only one article 28 aimed to evaluate the functional results after nerve-sparing posterolateral parametrial surgery, and the authors observed an increased risk of postoperative dyspareunia and sexual dysfunction. The FSFI sexual function score improved in the group without parametrial surgery, but not significantly. 28

The diagnosis of endometriosis was confirmed by histological examination of specimens removed during surgery in 15 studies. 13 14 15 17 18 20 21 24 25 26 28 30 31 33 34 Complementary surgical procedures for the treatment of endometriosis, including ureterolysis, adhesiolysis, salpingectomy and appendicectomy, were performed in ten articles. 13 14 15 16 21 24 27 28 29 33 Intraoperative or postoperative complications were reported in nine studies, 13 15 16 21 24 27 28 29 33 and the most common findings were transfusions caused by bleeding, transitory urinary retention, and bowel iatrogenic injury. Despite the complication rates reported, only one study 28 did not show a significant increase in sexual function after surgery.

The clinical treatment was an important point observed on this review. Some articles did not establish inclusion or exclusion criteria regarding the use of hormonal drug treatment associated with the procedure, but six studies 13 17 25 26 32 33 34 defined these criteria as In five studies, 17 25 26 32 34 hormonal treatment with gonadotropin-releasing hormone (GnRH) analogues and combined or isolated contraceptives were discontinued six months before the procedure, and two studies 25 32 did not reintroduce any type of hormonal treatment postoperatively. All studies presented an increase on sexual function, except, the one by Vercellini et al., 32 which did not show positive results on the SSRS after surgery.

One study 13 included a GnRH analogue preoperatively, and other studies included combined contraceptives preoperatively 31 33 and postoperatively. 33 Despite the differences regarding the hormonal treatment, the sexual function score on the SAQ and SHOW-Q improved postoperatively in two of these studies. 31 33

Dyspareunia, also called by some authors deep dyspareunia (DD) or pain during sexual intercourse, was assessed in 12 articles, 13 14 15 16 17 20 24 26 27 28 32 33 mainly through the Visual Analogue Scale (VAS) and the Numeric Rating Scale (NRS). Only Riiskjaer et al. 20 observed dyspareunia as an isolated finding, and evaluated it with its specific scale.

Three studies 17 27 34 identified a significant decrease in dyspareunia according to the NRS scale in all groups in the pre and postoperative comparison. The VAS was applied by the other articles to evaluate dyspareunia after surgery, and all articles reported a significant improvement in pain during intercourse after surgery, including progressive improvement in dyspareunia over time. Only one study 14 did not report a decrease in dyspareunia after 18 months of follow-up.

## Discussion

Due to its diverse origin, endometriosis presents great heterogeneity in terms of anatomical presentation and clinical manifestations, especially if associated with the complexity of multifactorial sexual aspects.

Qualitative and quantitative studies have shown that symptomatic endometriosis negatively affects female sexual function, causing discomfort, and they have analyzed these results through global scores. The isolated analysis of the domains of sexual function is unclear, and it is often not the main objective of studies, which limits a comprehensive assessment of sexual functioning. Therefore, the evidence in the literature lacks quality in terms of research design, diagnostic instruments, power of the study, or adjustment for confounding factors.

The present review helped expand the knowledge on the types of surgery performed to treat deep endometriosis, and we systematically analyzed the techniques used according to the location and staging of the disease, histopathological confirmation, nerve preservation, and the types of procedures performed for lesion resection.

The improvement in sexual function and dyspareunia after the surgical treatment in DIE patients was duly expressed by the authors of the studies reviewed. The laparoscopic surgery technique showed precision to treat DIE, in addition to the surgeons' experience. This statement is corroborated when there are positive results after surgeries, in addition to the correlation with other types of drug treatments.

All groups of patients classified according to the rAFS showed improvement in the quality of sexual life, especially those in classes IV and III; however it was not possible to identify the statistical relevance of the improvement in sexual function correlated with each group separately. 35 36

Autonomic, sympathetic, and parasympathetic nerves control the vessels in the genital region, and they are responsible for sexual satisfaction and lubrication. The nerve-sparing surgery for DIE is recommended to reduce patient morbidity. 37 However, 73.68% of the studies in this review did not perform the nerve-sparing surgery, neither did they find a direct correlation with female sexual function, as the literature. 29 38

The presence of DIE in the vagina and uterosacral ligaments is associated with impaired sexual function and dyspareunia. 39 The present review showed an improvement in female sexual function and postoperative dyspareunia despite the location of the endometriosis lesions, disease severity, and surgical treatment performed. We believe that the excision of inflammatory and angiogenic factors caused by DIE during surgery is the main factor for pain relief during sexual intercourse. Getting rid of feelings of fear and anguish caused by pain are also related to the improvement on other factors of sexual function.

In addition, the analysis related to deep dyspareunia still needs to be better developed, since the use of the NRS or probing alone is very simplistic compared with the psychological tests to distinguish deep dyspareunia from vulvodynia or vaginismus, which can also be triggered by chronic pelvic pain.

The lack of standardization among the questionnaires used to assess sexual function was a limiting factor in the present review, and it is due to the absence of an instrument capable of encompassing the complexity of DIE and its association with female sexual function. However, we were able to oppose some limiting factors found in the literature, such as follow-up time and questionnaire results. 40 We evaluated some studies with a follow-up longer than one year and with sexual function results demonstrated through the analysis of the domains involved in sexual response, such as arousal, satisfaction, pleasure and others.

## Conclusion

Highly-complex surgical approaches for the treatment of endometriosis have always been associated with the risk of complications arising from the excision of deep endometriotic lesions located mainly in the posterior vaginal fornix, rectal muscular layer, and inferior hypogastric plexus, which could worsen the patient's sexual quality of life and pain symptoms. Despite this, the present review demonstrated that radical surgeries for the treatment of DIE improved dyspareunia and sexual function, and they should be provided to women as a treatment alternative. Healthcare professionals should address the topic of sexual health in consultations with women with endometriosis because improvements following surgery can be expected. The present study not only demonstrates a significant reduction in dyspareunia symptoms, but it also shows that the resection of both minimal and extensive endometriotic disease causes major positive changes in sexual function.
